# A Reliable Method for the Evaluation of the Anaphylactoid Reaction Caused by Injectable Drugs

**DOI:** 10.3390/molecules21101352

**Published:** 2016-10-12

**Authors:** Fang Wang, Zebin Weng, Cunyu Li, Guoping Peng

**Affiliations:** 1 School of Pharmacy, Nanjing University of Chinese Medicine, Nanjing 210023, China; wangfang8875@163.com (F.W.); wengzebin@hotmail.com (Z.W.); licunyuok@163.com (C.L.); 2 Jiangsu Collaborative Innovation Center of Chinese Medicinal Resources Industrialization, Nanjing 210023, China

**Keywords:** anaphylactoid reaction, allergic reaction, inflammatory reaction, SC5b-9, IL-6

## Abstract

Adverse reactions of injectable drugs usually occur at first administration and are closely associated with the dosage and speed of injection. This phenomenon is correlated with the anaphylactoid reaction. However, up to now, study methods based on antigen detection have still not gained wide acceptance and single physiological indicators cannot be utilized to differentiate anaphylactoid reactions from allergic reactions and inflammatory reactions. In this study, a reliable method for the evaluation of anaphylactoid reactions caused by injectable drugs was established by using multiple physiological indicators. We used compound 48/80, ovalbumin and endotoxin as the sensitization agents to induce anaphylactoid, allergic and inflammatory reactions. Different experimental animals (guinea pig and nude rat) and different modes of administration (intramuscular, intravenous and intraperitoneal injection) and different times (15 min, 30 min and 60 min) were evaluated to optimize the study protocol. The results showed that the optimal way to achieve sensitization involved treating guinea pigs with the different agents by intravenous injection for 30 min. Further, seven related humoral factors including 5-HT, SC5b-9, Bb, C4d, IL-6, C3a and histamine were detected by HPLC analysis and ELISA assay to determine their expression level. The results showed that five of them, including 5-HT, SC5b-9, Bb, C4d and IL-6, displayed significant differences between anaphylactoid, allergic and inflammatory reactions, which indicated that their combination could be used to distinguish these three reactions. Then different injectable drugs were used to verify this method and the results showed that the chosen indicators exhibited good correlation with the anaphylactoid reaction which indicated that the established method was both practical and reliable. Our research provides a feasible method for the diagnosis of the serious adverse reactions caused by injectable drugs which could be used in the clinical practice.

## 1. Introduction

Drug-induced anaphylactoid reactions are pretty common in the clinic. Many drugs, especially injectable drugs such as anesthetics, contrast agents, anti-cancer drugs and herbal injections can induce varying degrees of anaphylactoid reaction which could be lethal. Herbal injection is one of the typical treatment options in China which exhibit excellent curative effects on cardiovascular diseases, respiratory diseases and cancer [1,2]. For example, Shuanghuanglian (SHL) injection is commonly used for the treatment of respiratory infections and has been one of the top selling herbal medicine products over the last decade. Yuxingcao (YXC) injection is an effective preparation for the treatment of respiratory diseases, gynecological inflammation and urinary tract infections. Although these herbal injections are widely used in clinical practice, the incidence of adverse reaction has increased in recent years [3]. Amongst the adverse reactions to Chinese medicines over the past decade, about 55.6% were caused by herbal injections [4]. Moreover, some Western medicine injections such as paclitaxel injections and levofloxacin injections can also cause anaphylactoid reactions [5,6]. Based on spontaneous adverse events reported by the US FDA, the incidence of anaphylactiod reaction after ciprofloxacin exposure has been estimated at 1.2 per 100,000 prescriptions in the United States [7].

It has long been believed that the adverse reactions of herbal injections were caused by anaphylaxis [8]. Anaphylaxis is a type I hypersensitivity reaction which occurs in response to encountered allergens. In this type of reaction, a sensitization and a triggering phase are distinguished. In the sensitization phase allergens are presented by dedicated antigen-presenting cells to Th2 lymphocytes, which then interact with B cells, antibody-producing lymphocytes, and help them in antibody switching and to produce allergen-specific IgE antibodies [9,10]. Secreted specific IgE circulates in the blood and binds to an IgE-specific receptor (to the high affinity receptor, called FcεRI) on the surface of other kinds of immune cells such as basophils and mast cells. These immune cells are both involved in the acute inflammatory response [11,12]. These allergen-specific IgE-bearing cells are now sensitized against the allergen and may trigger an allergic reaction upon the next contact with the allergen if at least 2 IgE antibodies on mast cells are bound to the allergen at the same time, a phenomenon called “bridging”, that leads to an allergen-specific mast cell degranulation and histamine release. However, most adverse reactions of herbal injections occur at first administration and are closely associated with the dosage. All these characteristics coincide with anaphylactoid reactions. At present, it is believed that there are two ways to cause anaphylactoid reactions: one is induced by the direct stimulation of mast cells or basophils; the other is caused by the activation of the complement system [13,14,15]. Stimulation of target cells induces anaphylactoid reactions by directly stimulating the G protein, which is a cellular protein involved in transmitting signals from a variety of stimuli outside a cell to its interior. Then a series of enzymes are activated leading to an increase in free calcium in the cytoplasm, inducing the degranulation of target cells and release of histamine and other mediators. In recent years, the complement system, especially the anaphylatoxins C3a and C5a was demonstrated to be involved in the pathogenetic mechanism of allergic diseases [16,17]. The anaphylatoxins C3a and C5a are generated immediately by the complement system when a sensitizing drug gets into the body. The C5a anaphylatoxin could induce mast cell degranulation and release of thromboxane A2 after binding to the surface receptors of mast cells. Then a series of allergic symptoms occur. In this process, the total complement activity is decreased while the content of terminal complement complex SC5b-9 is increased [18,19].

The initial presentation of anaphylactoid, allergic and inflammatory reactions is similar, and it is hard to differentiate anaphylactoid reactions from allergic reactions and sometimes even from inflammatory reactions simply based on their clinical presentation (Table 1). This can lead to clinical misdiagnosis and an enormous threat to the patient’s life. Therefore, it is very necessary to establish a practical and reliable method to diagnose anaphylactoid reactions quickly and accurately. In response to this need, in this study we use different animal models and injectable drugs to evaluate the expression of some representative physiological indexes in order to find the differences between anaphylactoid reactions, allergic reactions and inflammatory reactions.

## 2. Results

### 2.1. Anaphylactoid Reactions Provoked in Different Ways

**Figure 1:**
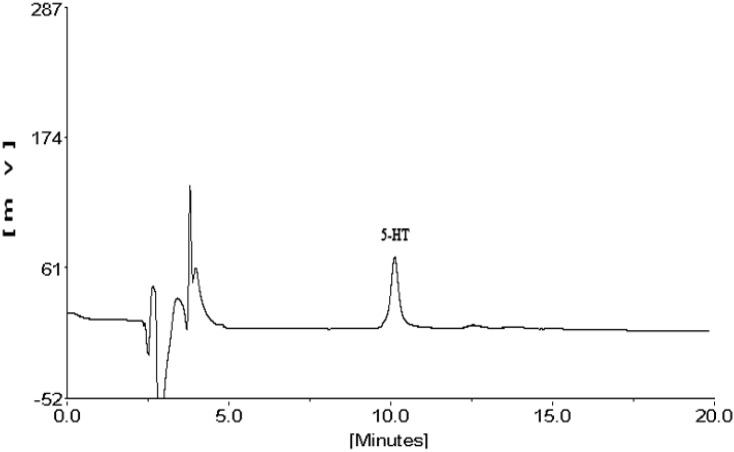
The HPLC chromatogram of serotonin.

Guinea pig and nude rat is usually utilized as the model animals in immunology studies, so in the present study, they were stimulated by compound 48/80 (C48/80) for different times (15 min, 30 min and 60 min), and the resulting 5-HT level in plasma was determined by HPLC analysis (Figure 1).

**Figure 2:**
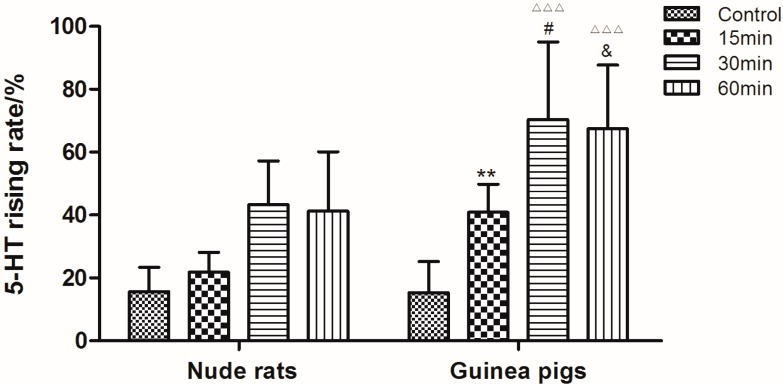
Rising levels of serotonin in guinea pigs’ and nude rats’ plasma after treatment with saline and C48/80 for different times (15, 30 and 60 min). ** *p* < 0.01 vs. Nude rats (15 min); **^#^** *p* < 0.05 vs. Nude rats (30 min); **^&^** *p* < 0.01 vs. Nude rats (60 min); ^△△△^ *p* < 0.01 vs. Control guinea pigs (*n* = 10).

**Figure 3:**
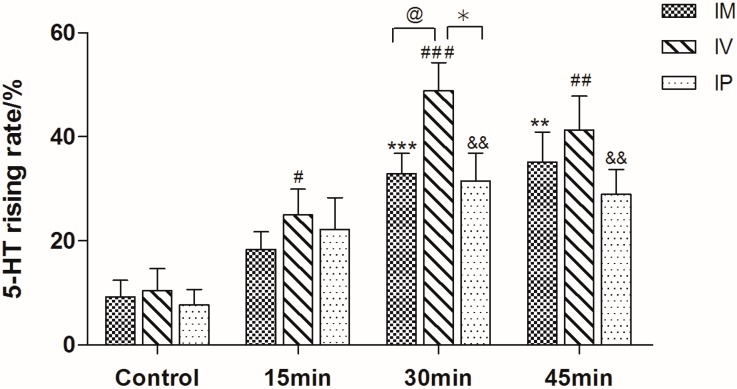
Rising levels of serotonin in guinea pig plasma after treatment in different ways (IM: intramuscular injection; IV: intravenous injection; IP: intraperitoneal injection). ** *p* < 0.01, *** *p* < 0.001 vs. IM control, **^#^** *p* < 0.05, **^##^** *p* < 0.01, **^###^** *p* < 0.001 vs. IV control, **^&&^** *p* < 0.01 vs. IP control, **^@^** *p* < 0.05 vs. IM, * *p* < 0.05 vs. IP group (*n* = 10).

As shown in Figure 2, the content of 5-HT in guinea pigs’ plasma was significant higher than in nude rat (*p* < 0.05). Therefore, guinea pig was chosen as the preferable animal model in the following research. For the purpose of investigating the optimized mode and time of stimulation, guinea pigs were treated with C48/80 through intramuscular injection (IM), intravenous injection (IV) and intraperitoneal injection (IP) for different times (15 min, 30 min and 60 min), respectively. As shown in Figure 3, the 5-HT levels of guinea pigs treated by IV were much higher than the other two modes (*p* < 0.05). Moreover, the results indicated that 30 min was the most appropriate stimulation time.

### 2.2. Selection of Physiological Index

It is well known that C48/80, ovalbumin (OVA) and endotoxin (LPS) can induce anaphylactoid reactions, allergic reactions and inflammatory reactions, respectively [20,21].

In order to distinguish these three pathological reactions, guinea pigs were stimulated by these three agents through IV and normal saline was used as the control. Blood samples were collected 30 min after treatment for the determination of some representative indicators of the three reactions (SC5b-9, 5-HT, Bb, C4d, histamine, C3a and IL-6).

**Figure 4:**
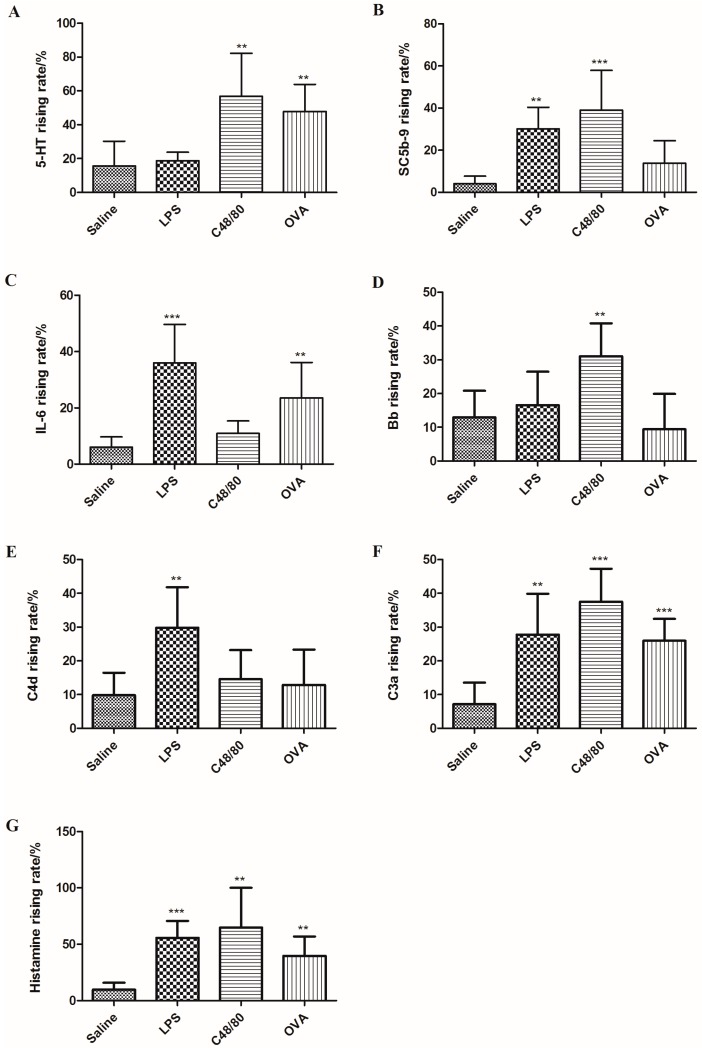
Rising rates of 5-HT, SC5b-9, IL-6, Bb, C4d, C3a and histamine in plasma after treated with C48/80, LPS and OVA. (**A**: 5-HT; **B**: SC5b-9; **C**: IL-6; **D**: Bb; **E**: C4d; **F**: C3a; **G**: histamine). ** *p* < 0.01, *** *p* < 0.001 vs. control (*n* = 10).

As shown in Figure 4, OVA could significantly increase 5-HT and IL-6 production. C48/80 could promote the secretion of 5-HT, SC5b-9 and Bb, while LPS administration resulted in increases of IL-6, SC5b-9 and C4d. In addition, the levels of C3a and histamine were significantly increased in all reactions. Therefore, five of the seven indicators including SC5b-9, 5-HT, Bb, C4d and IL-6 were chosen as the physiological indexes to distinguish between these three reactions. Changes of the investigated indicators are shown in Table 2. To distinguish between an anaphylactoid reaction and an allergic reaction, SC5b-9, IL-6, and Bb could be suitable indicators, while 5-HT, IL-6, Bb and C4d could be utilized to differentiate between anaphylactoid reactions and inflammatory reactions.

### 2.3. Measurement of Endotoxin in Each Injection

The dynamic turbidity method was performed using *Limulus* amebocyte lysate, an aqueous extract of blood cells from *Limulus polyphemus*, for the detection and quantification of endotoxin in SHL injection, YXC injection, paclitaxel injection, levofloxacin injection, diammonium glycyrrhizinate injection (negative control), toad venom injection and amiodarone hydrochloride injection. The detection range of *Limulus* amebocyte lysate is 0.01–10 EU/mL. As shown in Table 3, the content of endotoxin in each injection is far lower than the limit value.

### 2.4. Anaphylactoid Reaction Provoked by SHL Injection, YXC Injection, Paclitaxel Injection, Levofloxacin Injection and Diammonium Glycyrrhizinate Injection

**Figure 5:**
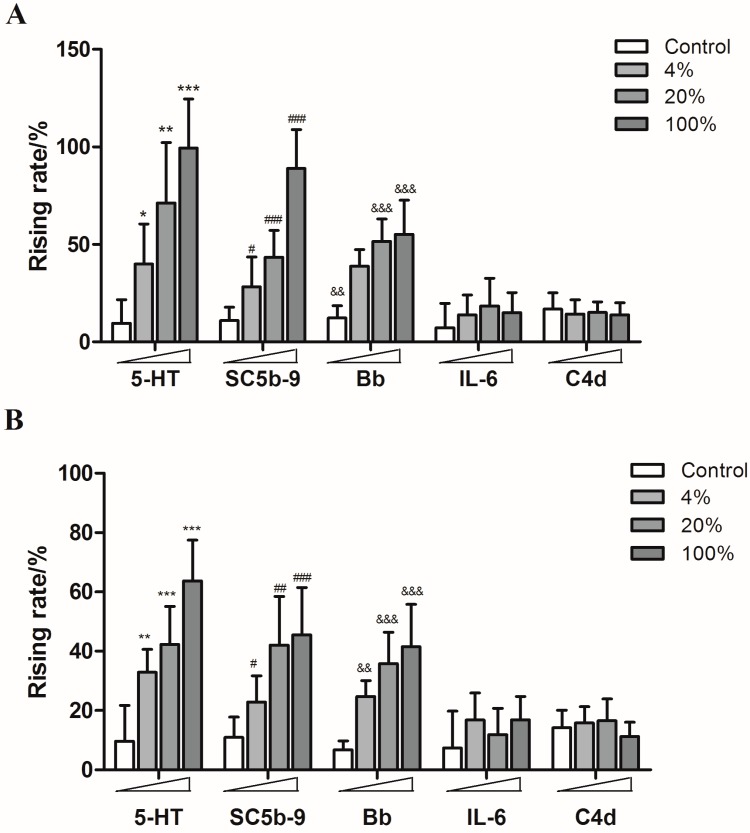
The rising rates of 5-HT, SC5b-9, Bb, IL-6, and C4d in animals’ plasmas after treating with different doses of SHL injection and YXC injection ((**A**): SHL injection; (**B**): YXC injection). * *p* < 0.05, ** *p* < 0.01, *** *p* < 0.001 vs. control; **^#^** *p* < 0.05, **^##^** *p* < 0.01, **^###^** *p* < 0.001 vs. control; **^&&^** *p* < 0.01, **^&&&^** *p* < 0.001 vs. control (*n* = 10).

**Figure 6:**
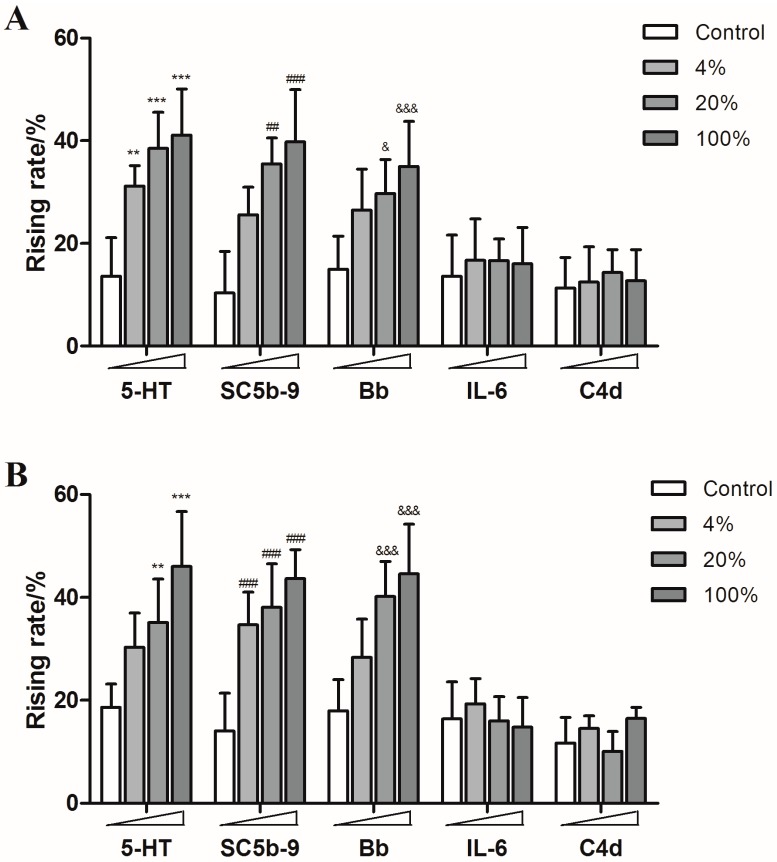

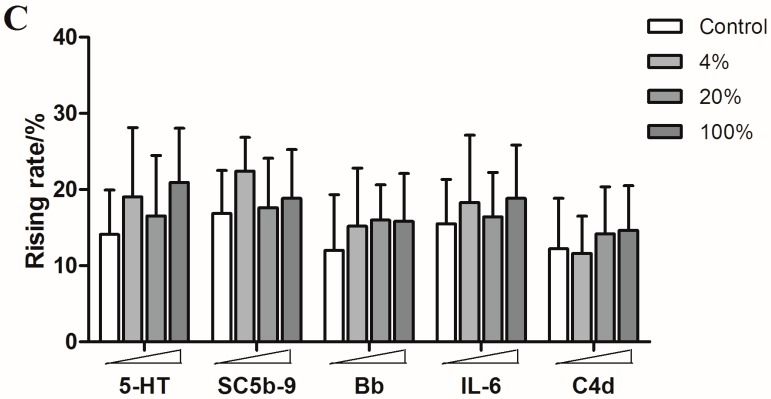
The rising rates of 5-HT, SC5b-9, Bb, IL-6, and C4d in animals’ plasmas after treating with different doses of paclitaxel injection, levofloxacin injection and diammonium glycyrrhizinate injection ((**A**): paclitaxel injection; (**B**): levofloxacin injection; (**C**): diammonium glycyrrhizinate injection). ** *p* < 0.01, *** *p* < 0.001 vs. control; **^##^** *p* < 0.01, **^###^** *p* < 0.001 vs. control; **^&^** *p* < 0.05, **^&&&^** *p* < 0.001 vs. control (*n* = 10).

Guinea pigs were treated with different concentrations of SHL injection, YXC injection, paclitaxel injection, levofloxacin injection and diammonium glycyrrhizinate injection, respectively. The content of 5-HT, SC5b-9 and Bb in plasma increased in a dose-dependent manner, while the production of IL-6 and C4d showed no significant increase, which indicated that both of SHL injection and YXC injection could cause anaphylactoid reactions (Figure 5) as well as the paclitaxel injection and levofloxacin injection which also caused the rise of 5-HT, SC5b-9 and Bb in plasma (Figure 6).

However, none of the five humoral factors increased significantly in the diammonium glycyrrhizinate injection groups (Figure 6). These results are consistent with clinical reports.

### 2.5. Inflammatory Reaction Provoked by Amiodarone Hydrochloride Injection and Toad Venom Injection

**Figure 7:**
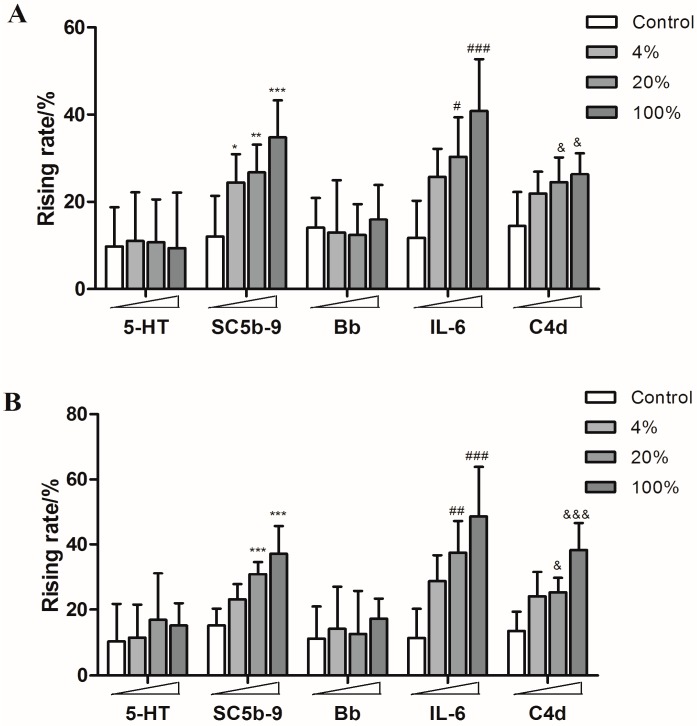
The rising rates of 5-HT, SC5b-9, Bb, IL-6, and C4d in animals’ plasmas after treating with different doses of amiodarone hydrochloride injection and toad venom injection ((**A**): amiodarone hydrochloride injection; (**B**): toad venom injection). * *p* < 0.05, ** *p* < 0.01, *** *p* < 0.001 vs. control; ^#^ *p* < 0.05, **^##^** *p* < 0.01, **^###^** *p* < 0.001 vs. control; **^&^** *p* < 0.05, **^&&&^** *p* < 0.001 vs. control (*n* = 10).

There are some reports about inflammatory reactions caused by amiodarone hydrochloride injection and toad venom injection [22,23], so they were also utilized to stimulate the guinea pigs in this study. As expected, both of them induced significant rises of SC5b-9, IL-6 and C4d levels (Figure 7).

## 3. Discussion

The anaphylactoid reaction presents with the same pathologic conditions and symptoms as the classical anaphylactic reaction and this affects the patient history gathering process, which may have a greater impact later on as a result. The clinical features of anaphylactoid, allergic and inflammatory reactions are similar. All of them can cause many autoimmune symptoms like spastic cough, conjunctivitis, urticaria and so on [24,25]. Although anaphylactoid reactions occur at the first contact while allergic reactions need generally a number of previous contacts to develop, it is impossible to differentiate them if the patients cannot provide a clear case history. So far, there is no reliable method to differentiate anaphylactoid reactions from allergic reactions or inflammatory reactions.

In this study, we have established a feasible method for the prediction of the anaphylactoid reactions to injectable drugs and differentiate them from allergic or inflammatory reactions by screening an animal model, optimizing the mode of drug administration and optimal selection of physiologic indexes. Two well-known herbal injections and two Western medicine injections which were reported to be allergenic were tested to verify this method. Diammonium glycyrrhizinate injection was also used as the negative control. The result showed that this method could distinguish between the anaphylactoid, allergic and inflammatory reactions of injectable drugs.

Compound 48/80 is a polymer produced by the condensation of *N*-methyl-*p*-methoxy-phenethylamine with formaldehyde. It promotes histamine release and is usually utilized to induce mast cell degranulation in biochemical research [14,26,27,28]. OVA and LPS are also used as positive controls in research on allergic reactions and inflammatory reactions [29,30]. In this study, compound 48/80, OVA and LPS were utilized as the positive drugs to induce anaphylactoid, allergic and inflammatory reactions, respectively. The results showed that the levels of 5-HT, SC5b-9, IL-6, Bb and C4d in guinea pigs’ plasma exhibited different responses in these three reactions. In anaphylactoid reactions, the levels of 5-HT, SC5b-9 and Bb were, significantly increased, while IL-6 and C4d levels showed almost no change. SC5b-9 is the end product of the complement system activation, so the determination of SC5b-9 levels can directly reflect the activation of the complement system [31]. Bb is the fragment of complement B factor after hydrolysis, which is a representative index of complement alternative pathway activation [32]. Consequently, we believe that the anaphylactoid reaction is likely to be produced by activating the complement system through alternative pathway activation. Inflammatory reactions induced increases of IL-6, SC5b-9 and C4d. C4d is the hydrolysate of C4 which is a representative indicator of the classical complement activation pathway [33]. The results indicated that both inflammatory reactions and anaphylactoid reactions can activate the complement system but through different pathways. In allergic reactions, 5-HT and IL-6 were found to be the apparently changed physiological indicators which were different from those of anaphylactoid reactions and inflammatory reactions. Therefore, we could distinguish anaphylactoid reactions, allergic reactions and inflammatory reactions by the combination of these five physiological index (5-HT, SC5b-9, IL-6, Bb and C4d) (Table 1). Our findings suggest a possible way to evaluate the allergenicity of the injectable drugs which may improve the safety of the clinical use of injectable drugs.

## 4. Experimental Section

### 4.1. Animals

Male Hartley guinea pigs and nude rats (Crl: NIH-Foxn1 rnu) weighing 250–350 g were purchased from the Laboratory Animal Center at Nanjing University of Chinese Medicine. All the animals were housed with a 12 h light/dark cycle at 22 °C and 55% ± 5% relative humidity and had free access to food and water. Animal experiment protocols in this study were carried out according to the guidelines of the Animal Care Committee of Nanjing University of Chinese Medicine.

### 4.2. Reagents

Compound 48/80 (C48/80) was purchased from Sigma-Aldrich (St. Louis, MO, USA; batch number is 073M4050V). Ovalbumin (OVA, batch number is 026K5448) was also purchased from Sigma-Aldrich. Endotoxin in the OVA was determined by *Limulus* Amebocyte Lysate assay (Xiamen BioEndo Technology Co., Ltd., Xiamen, China) and the content was less than 1 EU/mg. Serotonin (5-HT) was purchased from Sigma. *Shuanghuanglian* injection (SHL injection) and *Yuxincao* injection (YXC injection) were purchased from Harbin Jumbo Pharmaceutical Co., Ltd. (Harbin, China). Guinea pig-IL6 Elisa Kit, Guinea Pig-Bb Elisa Kit, Guinea pig-C3a Elisa Kit, Guinea pig-histamine Elisa Kit, Guinea pig-C4d Elisa Kit and Guinea pig-SC5b-9 Elisa Kit were all purchased from Amyjet Scientific, Inc. (London, UK).

### 4.3. Anaphylactoid Reactions Provoked by Different Ways

Animals were anesthetized with sodium pentobarbital through intraperitoneal injections (40 mg/kg). C48/80 was dissolved in normal saline at the concentration of 0.1 mg/mL. Guinea pigs and nude rats (*n* = 10/group) were stimulated by compound C48/80 (10 mg/kg) through intravenous injection. Blood sample A (1 mL) was collected 5 min before the stimulation through the carotid artery, and samples after the stimulation (blood sample B) were collected at different times (15 min, 30 min and 60 min). Guinea pigs were treated with C48/80 by three different ways: intramuscular injection, intravenous injection and intraperitoneal injection. The blood sample A and the blood sample B were collected through the animals’ carotid artery and the samples after the stimulation were collected at different times (15, 30 and 60 min). The serum was obtained through centrifugation (4000 rpm, 5 min) for the analysis of 5-HT.

### 4.4. Anaphylactoid Reactions, Anaphylactic Reactions and Inflammatory Reactions Provoked by Different Drugs

The guinea pigs used for anaphylactic reaction were pre-sensitized 21 days ahead the experiment. They were exposed to OVA 0.5 mL each time, q.o.d. for a total of three administrations. Then thirty normal guinea pigs and ten pre-sensitized guinea pigs were divided into four groups (normal saline, C48/80, LPS and OVA). C48/80, OVA and LPS were dissolved in normal saline at the concentration of 0.1 mg/mL and the guinea pigs were stimulated by intravenous injection (10 mg/kg). Blood sample A was collected 5 min before the stimulation through the carotid artery, and the samples after the stimulation(sample B) were collected at 30 min. Serum were obtained through centrifugation (4000 rpm, 5 min) for measurement of SC5b-9, 5-HT, Bb, C4d, histamine, C3a and IL-6.

### 4.5. Measurement of SC5b-9, 5-HT, Bb, C4d, Histamine, C3a and IL-6 in Animals’ Plasma

SC5b-9, Bb, C4d, histamine, C3a and IL-6 in the plasma samples were measured by enzyme-linked immunosorbent assay (ELISA) following the manufacturers’ instructions. The rising rate of SC5b-9, 5-HT, Bb, C4d, histamine, C3a and IL-6 was calculated according to the following equation:

(1) $$Rising\;rate\left(\text{\%}\right)=\frac{\left(valueB-valueA\right)}{valueA}\times 100\%$$

where *value B* is the amount of SC5b-9, 5-HT, Bb, C4d, histamine, C3a and IL-6 in blood sample B and *value A* is the amount of SC5b-9, 5-HT, Bb, C4d, histamine, C3a and IL-6 in blood sample A.

### 4.6. Measurement of Endotoxin in Different Injections

The endotoxin test was performed by the turbidity method. Briefly, an endotoxin standard substance (140 EU/mL) was diluted to 2.0, 0.5, 0.125, and 0.03125 EU/mL. A 100 μL portion of each dilution was mixed with the same volume of *Limulus* amebocyte lysate. The reaction mixtures were incubated at 37 °C and the optical density (OD) was simultaneously monitored. The time required to reach a designated turbidity (OD: 0.02) of the reaction mixtures was measured by endotoxin detector.

Analyses of the serial samples were performed using the least squares method. The relationship between endotoxin concentration and reaction time is as follows:

(2) logT=2.9641−0.1837logC

where *T* is the reaction time (s) and *C* is the endotoxin concentration (EU/mL). The related coefficient *r* = −0.9989.

### 4.7. Anaphylactoid Reactions Provoked by SHL Injection, YXC Injection, Paclitaxel Injection, Levofloxacin Injection and Diammonium Glycyrrhizinate Injection in Vivo

One hundred and sixty guinea pigs were randomly divided into sixteen groups. Guinea pigs were stimulated with different concentrations of SHL injection (4%, 20%, 100%), YXC injection (4%, 20%, 100%), paclitaxel injection (4%, 20%, 100%), levofloxacin injection (4%, 20%, 100%) and diammonium glycyrrhizinate injection (4%, 20%, 100%). Control group was treated with normal saline. Blood A were collected 5 min before the stimulation through the animals’ carotid artery, and the samples after the stimulation were collected at 30 min. Serum were obtained through centrifugation for the detection of SC5b-9, 5-HT, Bb, C4d and IL-6.

### 4.8. Inflammatory Reaction Provoked by Amiodarone Hydrochloride Injection and Toad Venom Injection

Seventy guinea pigs were randomly divided into seven groups. Guinea pigs were stimulated with different concentrations of amiodarone hydrochloride injection (4%, 20%, 100%), and toad venom injection (4%, 20%, 100%). Control group was treated with normal saline. Blood sample A (1 mL) were collected 5 min before the stimulation through the animals’ carotid artery, and the samples after the stimulation were collected at 30 min. Serum were obtained through centrifugation for the detection of SC5b-9, 5-HT, Bb, C4d and IL-6.

### 4.9. Statistical Analysis

Results were presented as mean ± SD. Experiments were conducted separately at least three times. Data were analyzed by one way ANOVA. Tukey’s multiple comparison test was made for multiple comparisons among all groups if overall *p* < 0.05. Probability (*p* value) of <0.05 was considered to be statistically significant.

## 5. Conclusions

In this study we have established a feasible method for the evaluation of the anaphylactoid reactions caused by injectable drugs. Such findings provide technical support for the treatment of the serious clinical adverse reactions of injectable drugs, and promote the normal development and use of injectable drugs.

## Figures and Tables

**Table 1:** Definitions and general features of different reactions.

Name	Definitions	General Features
Hypersensitivity	A state of altered reactivity where people react with an exaggerated immune response to a foreign agent. The term includes allergic and pseudo-allergic reactions, but sometimes it is also used synonymously with immune-mediated allergic reactions.	If it is used synonymously with true allergy, it requires a pre-sensitized (immune) state of the host. There are four basic types of allergic reactions: immediate hypersensitivity, cytotoxicity, immune-complex mediated reactions and delayed type hypersensitivity.
Type-I allergy or immediate type allergy	A type of allergic reaction induced by binding of allergen specific IgE antibodies to its high affinity receptor on mast cells (MC) resulting in antigen/allergen specific MC degranulation in pre-sensitized persons.	The reaction starts quickly, may be strong and fade quickly. It manifests as allergic rhinoconjunctivitis, allergic asthma or generalized urticaria or other organ manifestations.
Anaphylaxis	Anaphylaxis is simply the maximum variant of type-I allergy with systemic manifestation.	Anaphylaxis may begin within minutes or even seconds of exposure, can progress rapidly to airway constriction, generalized urticaria, intestinal and cardiovascular involvement. Without acute treatment it may end lethally.
Anaphylactoid reactions	Anaphylactoid reactions have no immunologic background and occur in a more dose-depend manner but require some individual predisposition too.	The clinical symptoms are similar to type-I reaction and therefore difficult to differentiate from IgE mediated Type I reactions.
Drug idiosyncrasy	Idiosyncrasy is an individual drug induced non-immunologic reaction and applies to genetically inherited enzyme defects involved in drug metabolism.	Idiosyncratic reactions occur therefore also in pre-disposed persons and may have similar features to allergic reactions.

**Table 2:** Changes of SC5b-9, 5-HT, Bb, C4d, histamine, C3a and IL-6 in anaphylactoid, anaphylactic and inflammatory reactions.

Humoral Factors	Anaphylactoid Reaction	Anaphylactic Reaction	Inflammatory Reaction
5-HT	+	+	−
SC5b-9	+	−	+
IL-6	−	+	+
Bb	+	−	−
C4d	−	−	+
C3a	+	+	+
histamine	+	+	+

“+” significantly increase, “−“ no significant increase.

**Table 3:** Endotoxin concentration in each injection.

Sample Name	Reaction Time (s)	Coefficient of Variation (%)	Recovery (%)	Contained (EU/mL)
4% SHL injection	2708	-	75.85	<0.01
	2729			
20% SHL injection	2139	0.36	114.34	0.0103
	2128			
100% SHL injection	1766	1.31	132.60	0.0274
	1799			
4% YXC injection	2698	-	109.78	<0.01
	2675			
20% YXC injection	2098	4.41	75.85	0.0095
	2233			
100% YXC injection	1714	1.75	114.34	0.0317
	1757			
4% paclitaxel injection	2757	-	120.69	<0.01
	2729			
20% paclitaxel injection	2478	-	114.54	<0.01
	2491			
100% paclitaxel injection	2209	1.68	93.93	0.0091
	2157			
4% levofloxacin injection	2641	-	105.88	<0.01
	2673			
20% levofloxacin injection	2765	-	84.25	<0.01
	2770			
100% levofloxacin injection	2174	1.08	117.77	0.0097
	2141			
4% diammonium glycyrrhizinate injection	2575	-	77.06	<0.01
	2589			
20% diammonium glycyrrhizinate injection	2187	2.20	78.62	0.0098
	2120			
100% diammonium glycyrrhizinate injection	1944	2.22	83.81	0.0157
	2006			
4% toad venom injection	1880	1.74	109.76	0.0195
	1926			
20% toad venom injection	2029	3.37	94.97	0.0162
	1934			
100% toad venom injection	2001	1.94	85.83	0.0141
	2056			
4% amiodarone hydrochloride injection	2209	1.98	118.82	<0.01
	2272			
20% amiodarone hydrochloride injection	2019	2.46	87.18	0.0133
	2090			
100% amiodarone hydrochloride injection	2069	-	88.96	0.0097
	2329			

## References

[1] Han R., Ye J.X., Quan L.H., Lin C.Y., Yang M., Liao Y.H. (2011). Evaluating pulmonary toxicity of Shuang–Huang–Lian in vitro and in vivo. J. Ethnopharmacol..

[2] Ren Y., Zhang P., Yan D., Wang J., Du X., Xiao X. (2011). A strategy for the detection of quality fluctuation of a Chinese herbal injection based on chemical fingerprinting combined with biological fingerprinting. J. Pharm. Biomed. Anal..

[3] Wang F., Li C., Zheng Y., Li Y., Peng G. (2015). Study on the anaphylactoid of three phenolic acids in Honeysuckle. J. Ethnopharmacol..

[4] Qian W., Yancong Z., Lijun X., Yangfang L. (2000). Retrospective Analysis of literature on ADRs of the Chinese material reported during 1990~1999. China Pharm..

[5] Vogel W.H. (2010). Infusion reactions: Diagnosis, assessment, and management. Clin. J. Oncol. Nurs..

[6] Smythe M., Cappelletty D. (2000). Anaphylactoid reaction to levofloxacin. Pharmacotherapy.

[7] Ho D.Y., Song J.C., Wang C.C. (2003). Anaphylactoid reaction to ciprofloxacin. Ann. Pharmacother..

[8] Ji K., Chen J., Li M., Liu Z., Xia L., Wang C. (2009). Comments on serious anaphylaxis caused by nine Chinese herbal injections used to treat common colds and upper respiratory tract infections. Regul. Toxicol. Pharmacol..

[9] Gould H.J., Sutton B.J. (2008). IgE in allergy and asthma today. Nat. Rev. Immunol..

[10] Alonso A., Jick S.S., Hernán M.A. (2006). Allergy histamine 1 receptor blockers, and the risk of multiple sclerosis. Neurology.

[11] Kraft S., Novak N. (2006). Fc receptors as determinants of allergic reactions. Trends Immunol..

[12] Maurer D., Fiebiger E., Reininger B., Ebner C., Petzelbauer P., Shi G.P. (1998). Fcε receptor I on dendritic cells delivers IgE-bound multivalent antigens into a cathepsin S-dependent pathway of MHC class II presentation. J. Immunol..

[13] Qiu S., Liu Z., Hou L., Li Y., Wang J., Wang H. (2013). Complement activation associated with polysorbate 80 in beagle dogs. Int. Immunopharmacol..

[14] Huang F., Zhang X., Zhang L., Li Q., Ni B., Zheng X. (2010). Mast cell degranulation induced by chlorogenic acid. Acta Pharmacol. Sin..

[15] Moghimi S.M., Hamad I., Andresen T.L., Jørgensen K., Szebeni J. (2006). Methylation of the phosphate oxygen moiety of phospholipid-methoxy (polyethylene glycol) conjugate prevents PEGylated liposome-mediated complement activation and anaphylatoxin production. FASEB J..

[16] Chenoweth D.E., Cooper S.W., Hugli T.E., Chenoweth R.W., Stewart E.H. (1981). Complement activation during cardiopulmonary bypass: Evidence for generation of C3a and C5a anaphylatoxins. N. Engl. J. Med..

[17] Huber-Lang M., Sarma J.V., Zetoune F.S., Rittirscha D., Lambrisc J.D., Drouind S.M. (2006). Generation of C5a in the absence of C3: A new complement activation pathway. Nat. Med..

[18] Weiszhár Z., Czúcz J., Révész C., Rosivall L., Szebeni J., Rozsnyay Z. (2012). Complement activation by polyethoxylated pharmaceutical surfactants: Cremophor-EL, Tween-80 and Tween-20. Eur. J. Pharm. Sci..

[19] Szebeni J., Baranyi L., Savay S., Lutz H.U., Jelezarova E., Bunger R., Alving C.R. (2000). The role of complement activation in hypersensitivity to pegylated liposomal doxorubicin (Doxil). J. Liposome Res..

[20] Rupa P., Mine Y. (2012). Oral immunotherapy with immunodominant T-cell epitope peptides alleviates allergic reactions in a Balb/c mouse model of egg allergy. Allergy.

[21] Thimmulappa R.K., Scollick C., Traore K., Yatesa M., Trusha M.A., Libyd K.T., Spornd M.B., Yamamoto M. (2006). Nrf2-dependent protection from LPS induced inflammatory response and mortality by CDDO-Imidazolide. Biochem. Biophys. Res. Commun..

[22] Li H. (2014). Care strategy damage in tissue and phlebophlogosis because of amiodarone injection. Med. Innov. China.

[23] Peng C., Sun G., Zou H., Jiang P., Wu Z., Zhang R., Nie X. (2010). Clinical Observation of the Effects of Phentolamine on Vascular Irritation Induced by Toad Venom Injection. China Pharm..

[24] Meiyu Z., Yikui L., Jia Z., Lianda L. (2009). Experimental study on anaphylactic and anaphylactoid reactions of houttuynia cordata injections. Chin. J. Mod. Appl. Pharm..

[25] Yanshuang F. (2002). Literature analysis of 187 allergic reactions associated with traditional Chinese medicines. J. Advers. Drug React..

[26] Rothschild A.M. (1970). Mechanisms of histamine release by compound 48/80. Br. J. Pharmacol..

[27] Choi Y.H., Yan G.H., Chai O.H., Song C.H. (2010). Inhibitory effects of curcumin on passive cutaneous anaphylactoid response and compound 48/80-induced mast cell activation. Anat. Cell Biol..

[28] Gibbs B.F., Schmutzler W., Vollrath I.B., Brosthardt P., Braam U., Wolff H.H. (1999). Ambroxol inhibits the release of histamine, leukotrienes and cytokines from human leukocytes and mast cells. Inflamm. Res..

[29] Al-Humadi N.H., Siegel P.D., Lewis D.M., Barger M.W., Ma J.Y.C., Weissman D.N. (2002). The effect of diesel exhaust particles (DEP) and carbon black (CB) on thiol changes in pulmonary ovalbumin allergic sensitized Brown Norway rats. Exp. Lung Res..

[30] Borovikova L.V., Ivanova S., Zhang M., Yang H., Botchkina G.I., Watkins L.R. (2000). Vagus nerve stimulation attenuates the systemic inflammatory response to LPS. Nature.

[31] Szebeni J., Alving C.R., Savay S., Barenholz Y., Priev A., Danino D. (2001). Formation of complement-activating particles in aqueous solutions of Taxol: Possible role in hypersensitivity reactions. Int. Immunopharmacol..

[32] Ballanti E., Perricone C., Muzio G., Kroeglera B., Chimentia M.S., Graceffaa D., Perriconea R. (2011). Role of the complement system in rheumatoid arthritis and psoriatic arthritis: Relationship with anti-TNF inhibitors. Autoimmun. Rev..

[33] Angioi A., Fervenza F.C., Sethi S., Zhang Y., Smith R.J., Murray D., Praet J.V., Pani A. (2016). Diagnosis of complement alternative pathway disorders. Kidney Int..

